# Development of the health promoting sports club—national audit tool

**DOI:** 10.1093/eurpub/ckac072

**Published:** 2022-08-26

**Authors:** Stacey Johnson, Anne Vuillemin, Aoife Lane, Kieran Dowd, Susanna Geidne, Sami Kokko, Alex Donaldson, Jan Seghers, Stephen Whiting, Aurélie Van Hoye

**Affiliations:** Université Côte ’Azur, LAMHESS, Nice, France; Université Côte ’Azur, LAMHESS, Nice, France; Technological University of the Shannon, Athlone, Ireland; Technological University of the Shannon, Athlone, Ireland; School of Health Sciences, Örebro University, Örebro, Sweden; Faculty of Sport and Health Sciences, University of Jyväskylä, Jyväskylä, Finland; Centre for Sport and Social Impact, La Trobe University, Melbourne, Australia; Department of Movement Sciences, KU Leuven, Leuven, Belgium; WHO European Office for Prevention and Control of Noncommunicable Diseases, Moscow, Russian Federation; Université de Lorraine, APEMAC, Nancy, France; Physical Education and Sport Sciences Department, University of Limerick, Ireland

## Abstract

**Background:**

Sports clubs have requested support from national governing authorities to invest in health promotion (HP), by developing policies, guidelines and dedicated funding. This article outlines the development of a national audit tool to review policies development and implementation to support HP in sports clubs.

**Methods:**

A five-step process was undertaken by an international project team: (i) a rapid literature review to identify items assessing policies in physical activity, HP and sports, (ii) a thematic analysis to categorize items, (iii) a Delphi method to analyze item relevance, country specificity, reformulation, validation and organization, (iv) face validity through an online survey and in-depth interviews with expert representatives on physical activity and sports and (v) audit tool finalization though project team consensus.

**Results:**

Eight sources were reviewed with 269 items identified. Items were coded into 25 categories with 3 broad themes: policies, actors and settings-based approach. The Delphi study extracted and refined 50 items and categorized them into 10 sections. After revisions from 22 surveys and 8 interviews, consensus was reached by the international project team on 41 items categorized into 11 sections: Role of ministry or department; Policies; Communication; Implementation and Dissemination; Evaluation and Measurement methods; Sub-national-level policies; Funding and Coordination; Participative approach; Actors and Stakeholders; National sporting events; Case studies and Implicated stakeholders.

**Conclusion:**

To progress HP in the sports club context it is necessary to understand existing national-level policies. This national audit tool will aid in monitoring and assessing national policies for health promoting sports clubs.

## Introduction

National health policies are strong indicators of a country’s support mechanisms for creating a healthier society.^1^ Health policies can be either formal or informal that ‘define priorities and action parameters to respond to health needs, available resources and political pressures’.^2^ Current policies rarely take into account the broader potential of settings-based health promotion (HP), where settings are considered as ‘places or social contexts where people engage in daily activities in which environmental, organizational and personal factors interact to affect health and well-being’.^2^ For example, several national policies exist to promote physical activity at the individual level through sport provision^3^^,^^4^ without considering the broader HP potential of sports clubs as a setting. Previous research highlights that one in five European countries had government-supported ‘Sport for Health’ programmes in place, while ∼1 in 3 had similar programs in place to promote physical activity.^5^ Additionally, the 2008 European Union (EU) Physical Activity Guidelines highlight the importance of sports clubs offering physical activity opportunities for the entire population, providing sports infrastructures within an acceptable distance (20 min by car or foot), and state the important role clubs can play in gender equality, social values and cultural development.^6^ Before this, HP through sport was mentioned in the EU White Paper on Sport^7^ and more recently, physical activity promotion specifically through sport is incorporated in the Global Action Plan on Physical Activity 2018–30.^3^

Organized sports clubs are ‘private, non-profit organizations formally independent of the public sector, including volunteer members and a democratic structure, having sports provisions as their main aim’.^8^ In Europe, sports clubs reach a wide audience with over 33% of the population participating in an organized sports club.^9^ Thus, sports clubs are active agents embedded in their society to promote citizenship, employment and inclusion.^10^ Sports clubs are unique settings because they can naturally promote the physical activity component of health,^11^ but they can also be informal venues to promote mental, social and well-being health outcomes.^12^ Since the early 2000s, researchers have explored the concept of HP in the sports club setting^13^^,^^14^ and in the process, developed a model and framework to guide the design, planning and implementation of HP interventions.^15^ The resulting health promoting sports club (HPSC) model and framework are based on a whole-system HPSC approach^16^ where HP is incorporated into daily sports club practices as well as all formal and informal policies.^17^ Recent research was undertaken to develop a settings-based model of the HPSC that includes both internal (participants, coaches, managers and the sports club as an organization) and external stakeholders (sports federations, public health agencies and government authorities) that influence a sports club’s ability to promote health.^15^ By incorporating multiple levels in the model, stakeholder roles for promoting health are clearly defined through four health determinants (economic, environmental, organizational and social) at each level. The HPSC model highlights the importance of government support, policies, education and financing, which are all essential elements of the HPSC approach. Previous research combined six case studies from five countries to establish the current state of HPSCs.^18^ Two major ‘themes’ emerged: (i) the investment in HP policies and practice by national sports organizations and sports clubs; and (ii), the inclusion of a wide range of stakeholders such as the community and parents of sports participants. Nevertheless, research to determine the extent of support provided to sports clubs to undertake HP, either directly through national levels or through affiliated sports federations, has been minimal. Although sports clubs can increase HP actions internally, top-down support is essential to provide guidance, policies, strategies and resources.^13^^,^^19^ A recent editorial described four types of HP in this setting: (i) as an outcome of the provided sport, (ii) sport used as a tool to create positive changes in a specific health determinant, (iii) HP in sport targeting a specific population or behaviour and (iv) the whole-system of sports incorporating all stakeholders and health determinants.^17^ Currently, little research has been conducted specifically on HP policies generated at the national level to support sports clubs to go beyond providing opportunities to participate in sport and physical activity, but to also promote health in a broader sense (physical, mental and social) and position themselves within the community to become agents of change.

In recent years, a number of frameworks and tools to monitor PA promotion at national levels, including the Health-Enhancing Physical Activity Policy Audit Tool (HEPA-PAT),^20^ the Comprehensive Analysis of Policy on Physical Activity framework^21^ and the Monitoring Framework for the EU Council Recommendation on HEPA Across Sectors^22^ have been established. Although useful, these tools are limited to PA promotion without considering the broader HP capacity of sports clubs.

This study describes the development of a national audit tool (NAT) that can be used to assess whether existing national policy frameworks support the implementation of the HPSC approach. The guiding question was: ‘How is HP in sports clubs supported through national-level policies and action plans?’ with several secondary questions including: (i) ‘what policies and support exist?’; (ii) ‘how are the policies implemented and disseminated?’; and (iii) ‘how are the policy results evaluated?’.

## Methods

A mixed-method, iterative study design^23^ was used to develop the HPSC-NAT. The project consisted of five primary steps: (i) a rapid literature review; (ii) indicator formulation and selection; (iii) online surveys; (iv) in-depth interviews; and (v) finalization of the HPSC-NAT. An international project team including nine researchers in sport, HP and public health from France, Ireland, Sweden, Finland, Belgium and Australia and one expert from the WHO Regional Office for Europe (WHO/Europe) was established. The project advanced over nine meetings from December 2020 through September 2021. The first meeting oriented the international project team towards the objective of creating the HPSC-NAT, the following four formulated sections and items of the HPSC-NAT for presentation to members of the EU HEPA focal points network,^5^ two follow-up meetings validated reformulations from online surveys and in-depth interviews, one meeting was conducted with the national EU HEPA focal points (national representatives appointed by EU Member States)^22^ to explain the audit tool and invite interested parties to take part in the online survey or interviews and a final meeting was held with a WHO/Europe representative to finalize the tool.

### Step 1: rapid literature review

To develop a preliminary list of items, a rapid literature review was conducted by two project team members. Rapid literature reviews allow for a targeted search based on researcher’s knowledge subject in literature and previously used data.^24^ The rapid literature review protocol included establishing criteria to search for primary data sources focussing on HP and/or physical activity policies, guidelines and analysis tools published or translated into English. The following eight sources were identified and reviewed for indicators: the English version of the CAPLA-Santé,^25^ three versions of the HEPA-PAT (English, Japanese and French),^20^ the HPSC strategies and indicator list,^15^ the e-PROSCeSS macro-level questionnaire,^26^ Sports Club for Health (SCforH) indicators and guidelines^27^ and the policy status review of HPSCs.^18^

### Step 2: indicator formulation and selection

Indicators identified during Step 1 were coded into categories in an excel spreadsheet and qualitative content analysis techniques were used to extract and interpret data.^28^ Indicators were analyzed to ensure relevance with the HPSC approach, remove country-specific indicators, identify and condense similar indicators and remove duplicates. Once indicators were cleaned within their respective categories, an initial rewording was made by two researchers (S.J. and A.V.H.). The wording of items was adjusted to reflect the HPSC approach rather than a single health behaviour. For example, the phrase ‘beyond sport participation’ was added to most items to emphasize that the HPSC approach has a vision to promote mental and social health rather than just focussing on physical health benefits of practicing sports. They were then presented to the project team as thematically categorized items and a modified Delphi method^29^ was used to select, discuss and refine the items during four iterative meetings. After each meeting, new versions were sent to project team members for additional comments and refinement. Categorized items and sections were refined until the team reached consensus on included sections, items and wording. In addition to the pre-selected items, team members could propose other relevant items to include in the audit tool and suggest the best section for inclusion. For example, adding items regarding implicated stakeholders who helped to complete the audit tool. The audit tool sections include all agreed upon items.

### Step 3: online surveys

Thirty physical activity experts, researchers and policy makers from the HEPA Europe Network, the SCforH network and the national EU HEPA focal points, from 30 countries were invited to respond to an online survey via *LimeSurvey* from April to May 2021 for face validity of the audit tool. Potential respondents were emailed an invitation and a detailed description of the project along with the participation link. Reminder emails were sent after 2 weeks. Surveys were anonymous and respondents could opt out at any time. Upon clicking the link, respondents answered demographic questions (gender, current work position, country and knowledge of the SCforH network or the HEPA Europe network). Each item from the audit tool was presented in its respective section. Participants were asked to respond using a 4-point Likert scale (totally agree, agree, disagree and totally disagree) to the following section questions: ‘You have understood the section’, ‘You think the section is clearly formulated’, ‘You know how to respond to the section’, ‘You think the section can be completed for your country’, ‘You can find the necessary information to this section for your country’, ‘You think the section is relevant to review policies supporting HP in sports clubs’. At the end of each section, participants were given the opportunity to include additional comments: ‘Do you have any reformulation suggestions or remarks for a specific question in this section or for the whole section in general’. They were not asked to complete the audit tool. Respondents were then asked to rank each section in order of importance to evaluate HPSC policies. Quantitative data were analyzed with descriptive statistics through Excel Quick statistics and qualitative data were analyzed by two researchers (A.V.H. and S.J.) through qualitative data coding methods.^30^ Section ranking was analyzed by a weighted scoring method whereby each time a section was ranked as first (most important) it was assigned 10 points, 2nd rankings received 9 points, 3rd received 8 points etc., until finally, 10th place received 1 point. The mean scores were used as placement for rankings (highest mean in first place).^12^^,^^31^ If sections had the same mean score, minimum and maximum scores were compared with choose the appropriate placement.

### Step 4: in-depth interviews

Twenty-eight national EU HEPA focal points were briefed on the HPSC approach and audit tool during a dedicated meeting before being invited to participate in an in-depth interview. Invitation emails were then sent out to the focal points in early May 2021. Individual interviews were conducted from late May through early September 2021 and lasted ∼1.5 h each. A working copy of the HPSC-NAT was sent 1 week in advance of each interview so participants could review the content. During the interview, participants were asked the same demographic questions as in Step 3. Then, the interviewer (S.J.) went through each section and asked if each item was easily understood, any suggested changes and comments. Notes from each interview were detailed and coded in an excel spreadsheet. Once all interviews were completed, qualitative data analysis was conducted by two researchers (S.J. and A.V.H.).^28^ Suggested changes were considered by the international project team if at least two participants made the same or similar comment as noted in the coding process.

### Step 5: HPSC-NAT finalization

The project team met for two finalization meetings where suggestions from the survey and in-depth interviews were discussed and adjustments to sections or items were made with majority consensus.

## Results

### Step 1: rapid literature review

From the 8 primary sources examined, 269 total items were identified: 17 in the CAPLA-Santé,^25^ 76 from the three HEPA-PAT versions,^20^ 18 from HPSC strategies,^15^ 26 from the e-PROSCeSS questionnaire,^26^ 45 from the SCforH guidelines^27^ and 84 from the policy status review of HPSCs.^18^ Three extra items were added by the project team to gauge a country’s knowledge on HP; e.g.: ‘Is HP a well-known or well-understood concept at the national or local level?’ and ‘Is settings-based HP used when planning and developing policies?’.

### Step 2: indicator formulation and selection

**Figure 1.**
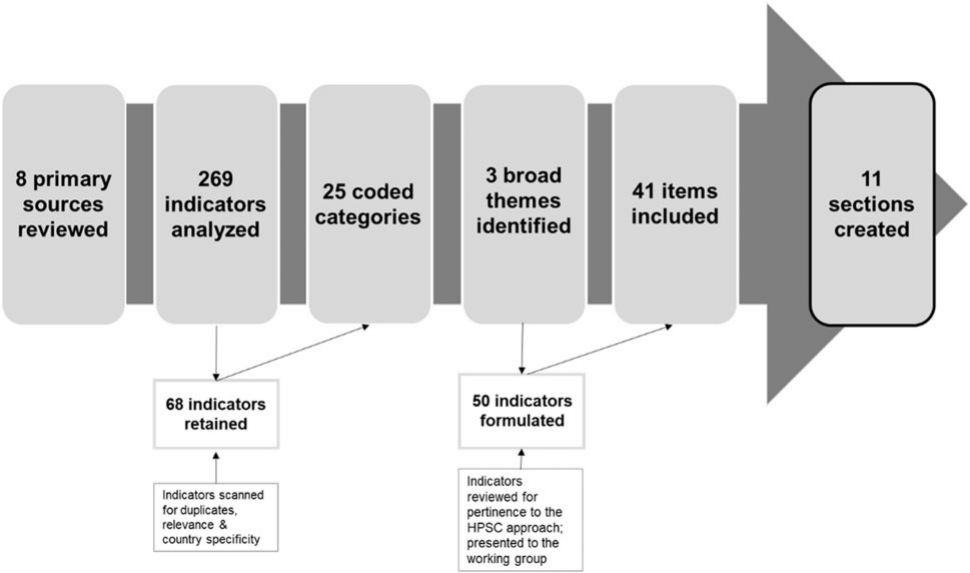
Process of developing the HPSC-NAT

Once the 269 items from Step 1 were analyzed, 68 items remained (see figure 1) and were coded into 25 categories (i.e. policy, funding, support, network, orientation, partners etc.) with three broad themes emerging: policy, actors and settings-based approach. Categories and items were reviewed for relevance to the HPSC approach with 50 items being retained. These were developed into 25 primary items with 30 follow-up items to present to the project team for discussion, removal, re-wording and ordering into sections based on categories. For example, because the term ‘health promotion’ is not widely understood by sports club stakeholders and potential respondents to the NAT, the item: ‘Which national ministries target HP policies in the sports sector’ was re-worded: ‘Does your country have a national ministry (or department) that is mainly responsible for supporting sports clubs to address health topics (social, mental, physical health; well-being; sustainability etc.) beyond sport participation?; If yes, please indicate which one(s)’. Some section names were modified and some items were removed due to comprehension difficulties. For example, ‘Are HP determinants acknowledged and understood at the national, regional, local level?’ was removed because it was decided that many respondents might not understand health determinants or implementation strategies to improve them. Consensus was reached from the project team on 11 sections after four iterative meetings. Sections included: Role of ministry or department; Policies; Communication; Implementation and Dissemination; Evaluation and Measurement methods; Sub-national-level policies; Funding and Coordination; Participative approach; Actors and Stakeholders; National sporting events; Case studies and HPSC-NAT implicated stakeholders (see table 1 detailing the evolution from original items to the finalized HPSC-NAT).

**Table 1:** Indicators formulated into the HPSC-NAT

Thematic	Category	Original proposed item	Team reformulations/additions	Section	Finalized HPSC-NAT
Policy	Ministry implication	Which ministries/departments target sports clubs as a vehicle to address other issues beyond sport participation? (health, well-being, social justice, sustainability etc.)	Does your country have a national ministry (or department) whose main responsibility is to support sports clubs to address health topics (social, mental, physical health; well-being; sustainability etc.) beyond sport participation? (note: support could include policies, strategies, action plans etc.^a^)	Role of ministry or department	Does your country have a national ministry (or department) that is mainly responsible for supporting sports clubs to address health topics (social, mental, physical health; well-being; sustainability etc.) beyond sport participation? (note: support could include policies, strategies, action plans etc.^a^)
			Are any other Ministries or departments implicated in supporting sports clubs to address health topics (social, mental, physical health; well-being; sustainability etc.) beyond sport participation?^b^		Are any other ministries or departments involved in supporting sports clubs to address health topics (social, mental, physical health; well-being; sustainability etc.) beyond sport participation?
	Policies	What policies exist at a national level to support HP as a goal in sports clubs?	Does your country have any national policies that guide sports clubs to address health topics (social, mental, physical health; well-being; sustainability etc.) beyond sport participation?	Policies	Does your country have any national policies that guide or request sports clubs to address health topics (social, mental, physical health; well-being; sustainability etc.) beyond sport participation?
			If yes, what is the title, date, issuing body and if available, the website:^b^		If yes, what is the title, date, issuing body and if available, the website and briefly describe the content and objectives of the policy in regards to HP within sports clubs
	Policies	Are there distinct HP policies for different goals? (injury prevention, inclusion and PA)	Are any of the following health topics targeted in the policy: Injury prevention, Social inclusion, Healthy diet, Physical activity, Sleep, Mental health, Alcohol, Tobacco, Sustainable development, Gender equity and Other		Are any of the following specific health topics targeted (check all that apply): Alcohol, Doping, Gender equality, Healthy diet, Injury prevention, Mental health, Physical activity, Safety, Sleep, Social inclusion, Sustainable development, Tobacco, Unhealthy sponsorship and Other
	Policies	Are there distinct HP policies for different target populations? (children, chronic diseases, youth, disabled, seniors etc.)	For this policy, list (check) the target population(s): Early years, Children/Youth, Families, Students, Adults, Seniors, Vulnerable population, People with disabilities, Employees, Women, Sedentary people, Low socioeconomic populations, Migrant populations, General population and Other		For this policy, check the target population(s) (check all apply): Adults (18–64 years), Athletes, Children/Youth (5–12 years), Early years (0–4 years), Ethnic/indigenous populations, Families, Low socioeconomic populations, Men, Migrant populations, People with disabilities, Sedentary people, Seniors (65+ years), Students (13–17 years), Vulnerable/at risk populations, Women, Workforce/employee and Other
	Policies	What materials did your organization refer to in order to formulate the guidelines? (SCforH, Exercise and PA reference for HP, WHO Global recommendations on PA for health etc.)	Did the ministry consider the following documents when formulating the policy: HEPA-PAT, Sports Clubs for Health guidelines, Global strategy on diet, physical activity and health, Global action plan for physical activity, Global action plan for the prevention and control of non-communicable diseases 2013–20, EU White Paper on Sport and Other		Did the ministry or issuing body consider any of the following documents when formulating this policy (check all that apply): EU Physical Activity Guidelines, WHO’s HEPA-PAT, SCforH, WHO’s Global strategy on diet, physical activity and health, Global action plan for the prevention and control of non-communicable diseases 2013–20, Physical activity strategy for the WHO European Region 2016–25, WHO’s Global action plan for physical activity 2018–30, EU White Paper on Sport and Other
			Does your country have any secondary national policies that guide sports clubs to address health topics (social, mental, physical health; well-being; sustainability etc.) beyond sport participation?		Does your country have national policies in which guiding sports clubs to address health topics (social, mental, physical health; well-being; sustainability etc.) beyond sport participation is a secondary objective?
	Communication	Are there specific communication strategies used?	Please briefly describe below how the previously cited policies are disseminated/communicated to the subnational levels? Provide the dissemination activities (poster, website, mail, labelling) and target (sub-national sport politician, general population etc.).	Communication, Implementation and Dissemination	Does your country have a national communication strategy/plan to disseminate the previously cited policies (Section 2) to sub-national levels and/or directly to sports clubs?
			Please briefly describe the strategies/action plans at the subnational level, who are the actors implicated, what is included?^b^		Please briefly describe how the previously cited policies (Section 2) are disseminated/communicated to the sub-national levels? List the dissemination activities (poster, website, mail, labeling) and respective target groups to receive the policy information (subnational sport politician, general population etc.)
	Communication	How is knowledge on HPSC communicated or disseminated (e.g. a developed communication plan)?	Does the Ministry (or department) in charge of the communication/dissemination engage directly with sports clubs? If so, briefly describe how:		Does the Ministry or department in charge of the communication/dissemination engage directly with sports clubs? If so, briefly describe how:
	Implementation strategies	What strategies are implemented to drive and sustain HP activities?	What are the top three actions used to support implementation of the policy within grassroot sports clubs?		In your opinion, what are the top three actions used to support implementation of these policies at the subnational level?
	Implementation strategies	Are HP benefits relayed to SC and partners? (diverse membership, establish identity in the community, increased volunteers, increased support for HP)^a^			
	Evaluation	Are HP policies and activities evaluated? (internal, external and national indicators of health or PA)	Are any of the previously cited policies evaluated?	Evaluation and Measurement methods	Is the implementation of any of the previously cited policies evaluated?
	Evaluation	Who are the experts to evaluate, how is the evaluation developed?	Please state how the implementation of each main policy was evaluated, what was evaluated, collection methods, results summary, how were results used. Report title, Year published, Web link, Responsible party for evaluation, Summary and Use of results		If so, please explain how the implementation of each main policy from Section 2 was evaluated: what was evaluated, data collection methods, results summary, how results were used: Report title, Year published, Web link, Data collection methods, Responsible party for evaluation, Data evaluated, Summary of evaluation results and Use of the results
	Evaluation	Are any measurements used (i.e. surveys) to monitor how HPSC is present among sports clubs?	Does your country monitor actions on health topics within sports clubs? Refer to the list from Section 2 (i.e. injury etc.)		Does your country monitor actions on health topics [refer to Section 2 list (i.e. injury risk, safety etc.)] within sports clubs, beyond sport participation?
			What measurements or surveys are used to monitor how to support sports clubs to address health topics (social, mental, physical health; well-being; sustainability etc.) beyond sport participation?		What measurements or surveys are used to monitor the actions on these health topics?
			Please briefly describe (50–100 words) how information gathered helps to plan future policies:		Please briefly describe (e.g. 50–100 words) how the gathered information helps to plan future policies:
	Sub-national levels	At what levels are the guidelines implemented in sports clubs in your country? (national, regional and community levels)	Are there any regional, local or sports organization policies implemented in your country to address health topics (social, mental, physical health; well-being; sustainability etc.) in sports clubs?	Sub-national-level policies	Are any regional, local or sports organization/association policies implemented in your country to address health topics (social, mental, physical health; well-being; sustainability etc.) in sports clubs?
	Sub-national levels		Please provide three primary examples: Title, Timeframe, Issuing body, Website and Brief description		Please provide three primary examples: Title, Timeframe, Issuing body, Website and Brief description
			Are any regional or local strategies implemented in your country to address health topics (social, mental, physical health; well-being; sustainability etc.) in sports clubs?		Are any regional or local strategies (different from policies) implemented in your country to address health topics (social, mental, physical health; well-being; sustainability etc.) in sports clubs?
	Funding	How is the financial support for HPSC attained? (fundraising, national government grants, sports association, public funding, political support and international grants)	Does your country have specific funding opportunities to support sports clubs to address health topics (social, mental, physical health; well-being; sustainability etc.) beyond sport participation?	Funding and Coordination	Does your country have specific funding opportunities to support sports clubs to address health topics (social, mental, physical health; well-being; sustainability etc.) beyond sport participation?
			Briefly describe the funding sources and the process sports clubs use to access this funding. Is it national funding, subnational funding, recurring or one-time? Source, Level, Amount, Recurrent and Comments		Briefly describe the funding sources (i.e. state or national budgets, EU funding etc.) and the process sports club use to access this funding. Is it national level funding, sub-national level funding, recurring or one-time? Source, Level, Amount, Recurrent and Comments
	Coordination/Network	Has a specific coordinating mechanism (e.g. working group, coordinating institution etc.) been developed for HPSC in your country?	Is there cross-sectoral collaboration to align policies or strategies to guide sports clubs to address health topics (social, mental, physical health; well-being; sustainability etc.) beyond sport participation?		Is there cross-sectoral collaboration to align policies or strategies to guide sports clubs to address health topics (social, mental, physical health; well-being; sustainability etc.) beyond sport participation?
	Coordination/Network	Are there links made within and between sectors to align policies for promoting health?			Please briefly describe who is involved in the collaboration, who coordinates the efforts and how the collaboration functions
Settings-based approach	Bottom-up	Does your country use a participative approach or bottom-up approach when framing policies?	Does your country use a participative approach (consultative process) when framing national policies for sports clubs to address health topics (social, mental, physical health; well-being; sustainability etc.) beyond sports participation?	Participative approach	Does your country use a participative approach (consultative process) when framing national policies for sports clubs to address health topics (social, mental, physical health; well-being; sustainability etc.) beyond sports participation? Briefly outline how relevant stakeholders and organizations are included
	Needs assessment	Are there any perceived motives or barriers to HPSC (strengths/weaknesses)?	What are the three main challenges for sports clubs to develop policies to address health topics (social, mental, physical health; well-being; sustainability etc.) beyond sports participation?		In your opinion, what are the three main challenges that sports clubs face when developing or implementing policies or actions to address health topics (social, mental, physical health; well-being; sustainability etc.) beyond sports participation?
	Needs assessment	Are there any perceived motives or barriers to HPSC (strengths/weaknesses)?	What are the three main motives for sports clubs to develop policies to address health topics (social, mental, physical health; well-being; sustainability etc.) beyond sports participation?		In your opinion, what are the three main motives for sports clubs to develop or implement policies or actions to address health topics (social, mental, physical health; well-being; sustainability etc.) beyond sports participation?
**Actors**	Partnerships and support	Were partnership created with other sectors/actors to promote health in sports clubs?^a^			
	Partnerships and support	Do sports clubs have defined roles, responsibilities and expectations while working with partners?	Are sports clubs encouraged to create partnerships with other sectors?	Actors/Stakeholders	Do national ministries or departments provide guidance for sports clubs to create partnerships with other sectors to support HP actions? Briefly describe how they are guided
	Stakeholders	Are there specific stakeholders who support HP in sports clubs?	Do other private companies, charities, advocacy groups, academia, the scientific community or non-government organization (NGOs) support sports clubs to address health topics (social, mental, physical health; well-being; sustainability etc.) beyond sport participation?		Do private companies, charities, advocacy groups, academia, the scientific community or NGOs support sports clubs to address health topics (social, mental, physical health; well-being; sustainability etc.) beyond sport participation? If yes, which organizations
	Education	In your country, is there any training provided focussing on the HPSCs approach?	In your country, is any training provided which focuses on social, mental, physical health or well-being and sustainability in sports clubs? Type of training, Who receives training, How is it provided, Level (national or subnational) and Training details		In your country, are any education opportunities provided by any level of government focussed on social, mental, physical health or well-being and sustainability in sports clubs? If yes, please describe the training
	Event organization	Is HP considered in sports event organization?	Are health topics (social, mental, physical health; well-being; sustainability etc.) taken into account when planning national sporting events?	National sporting events	Are health topics (social, mental, physical health; well-being; sustainability etc.) considered when planning national or sub-national sporting events such as those overseen by governmental sports departments/agencies (i.e. national Olympic committees, sports federations etc.)? If yes, please describe how they are taken into account during the planning stage
	Case studies	Are there any examples you can give of exemplar HPSCs in your country?	Are there any examples you can give of programs which encourage sports clubs to address other health topics (social, mental, physical health; well-being; sustainability etc.) beyond sport participation?	Case studies	Are there any examples you can give of programmes which encourage sports clubs to address other health topics (social, mental, physical health; well-being; sustainability etc.) beyond sport participation? Briefly describe the programmes: Programme name, Participants involved, Levels involved (national, regional, provincial and municipal), Programme description
			Leader of the HPSC-NAT completion process: Name, Institute and Contact details	HPSC-NAT implicated stakeholders	Leader of the HPSC-NAT completion process: Name, Institution and Contact details
			Other team members of HPSC-NAT completion process: Name and Institute/Organization		Other team members of HPSC-NAT completion process: Name and Institute/Organization
			Explain the completion process: Month/year, Main steps and Comments		Explain the completion process: Month/year, Main steps and Comments
			Please list the consulted experts for input on the HPSC-NAT: Contact person and Institute/Organization		Please list the consulted experts for input on the HPSC-NAT: Contact person and Institute/Organization

a Item deleted.

b Item added by the project team.

### Step 3: online surveys

**Figure 2.**
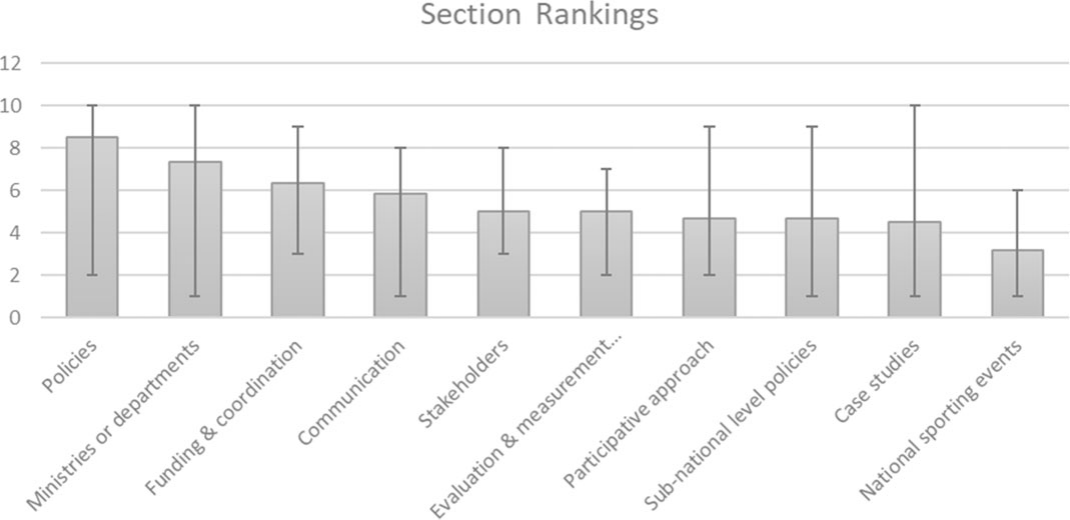
Section rankings by order of importance. Grey bars are mean rankings for each section. Range bars indicate the minimum and maximum rankings for each section

Twenty-two participants responded to the survey (see table 2). Based on the Likert scale, all section descriptions and included items were understood. Through qualitative data collection, minor modifications were suggested, such as separating options for different ministries in Section 1 (i.e. separate the ministry of Education and Culture into two options) or replacing the word ‘transport’ with ‘transportation’. In Section 2, several respondents suggested adding ‘Unhealthy sponsorships’ to the checklist for policy objectives and adding age ranges for target populations. Respondents suggested adding the phrase ‘in your opinion’ when referring to actions supporting HP in sports clubs in Section 3. Two respondents mentioned that they would have to contact other people to obtain answers about funding sources and coordination methods in Section 7. For this reason, in Section 11 the project team added an item requesting information about additional people contacted to complete the audit tool. After adjusting for non-responses, the top three sections ranked in order of importance to evaluate HP policy support in sports club were: Policies, Ministries or departments and Communication (see figure 2).

**Table 2:** Demographic details for online surveys and in-depth interviews

	Online survey	Interviews
	Respondents (*n* = 22)	Respondents (*n* = 8)
Gender		
Female	3	5
Male	5	3
No answer	14	
Current position		
NGO	2	3
Public organization	0	3
Private organization	0	
Academia	5	2
Other	1	
No answer	11	
Country represented		
Australia	1	
Bulgaria		1
Croatia	1	1
Czech Republic	1	
Finland		2
France		1
Germany	1	
Hungary		1
Ireland	2	
The Netherlands	1	
Portugal		2
Slovenia	1	
No answer	14	

### Step 4: in-depth interviews

In total 8 of the 28 EU HEPA focal points participated in an in-depth interview (see table 2). One major comment received during several interviews was that many of the responses to items would be ‘no’ because they had not implemented HEPA policies yet—‘the HPSC approach is not yet known therefore, policies regarding this are far off in the future’. Several of the responding countries mentioned that their government was still focussed on implementing physical activity policies in settings such as sports clubs. Therefore, their ministries were not in a position to focus on the broad vision of promoting health (i.e. social inclusion, nutrition, civic duty) beyond the immediate benefits of participating in physical activity through sport. In addition, several focal points commented that ‘the government is more focussed on competitive sport and sustaining funding for sports federations rather than providing support for local-level sports clubs’. Several focal points mentioned that it will ‘take time to differentiate HPSC from HEPA and SCforH since these two are already well-known’. Minor changes were suggested to the formulation of some items in Sections 2 and 11. In addition, two focal points mentioned that the tool would need to be available in the country’s native language as many words, such as ‘beyond’ do not have a precise translation or the word ‘training’ may be misconstrued and would need to be replaced with ‘education’ in his/her native language.

### Step 5: HPSC-NAT finalization

Two final project team meetings were conducted to discuss the suggested changes from the online surveys and in-depth interviews. As no major changes were requested by the focal points, consensus was obtained on the minor changes required (see table 1). The timeline for completing the HPSC-NAT was also defined as 3–6 months, based on the seven-steps described in the tool’s introduction. It is also suggested that the process of completing the tool be led by a national representative from the sport or health sector and repeated every 2–3 years.

## Discussion

This NAT has been designed to enable a comprehensive review of existing national policies focussed on supporting sports clubs to address health topics beyond sports participation. It is hoped it will act as a springboard to develop inter-sectoral approaches, policies and action plans to encourage HPSC. An international project team undertook the process of designing the audit tool through a five-step iterative approach incorporating both qualitative and quantitative data collection methods. An initial 269 items were identified. With the help of experts in the fields of physical activity and sports, 41 items were validated and classified into 11 sections detailing specific policies that support the HPSC approach. The tool was finalized after nine meetings. Due to several comments highlighting potential issues with obtaining the information to complete the audit tool, a final section regarding implicated stakeholders was added. This will aid in future development of the HPSC-NAT by showing differences in collection methods, adding additional options or refining certain items.

As the HPSC approach is a relatively recent development, many countries are still unfamiliar with the concept and the promotion of health beyond PA is still not commonly integrated in sports club settings. Although the call of health-focussed policies in sports clubs is not new,^6^ even HEPA and SCforH indicators are not fully employed at national levels.^32^ Therefore, national policies can aid sports clubs to develop their HP actions and further embedded into local communities. When speaking about sports clubs, most EU HEPA focal points emphasized that governments focus on the core business of sports provision and competition rather than the more complex concept of HP which is the basis of the HPSC approach and has been previously reported.^8^ When developing related policies, their focus is on promoting HEPA in various settings. For example, several focal points mentioned that their government ministries are still trying to implement physical activity policies and implicate sports clubs in this process. Additionally, although many focal points were aware of SCforH, they had little understanding of the HPSC approach including its promotion of physical, mental and social health and well-being beyond focussing on the health-enhancing benefits of a particular sport which is one of the core premises of SCforH.^27^ Therefore, HPSC is a settings-based approach focussing on HP within the specific setting of sports clubs and is therefore differentiated from HEPA which specifically focuses on promoting physical activity in all settings. Both of these are distinct from SCforH which focuses on health benefits sports participants gain from participating in a particular sport or sports club.

Previous research acknowledged that sports clubs need support to develop HP, being limited in resources due to their voluntary nature,^19^ and that external stakeholders such as national governments and affiliated sports federations could provide funding, guidelines, training and advocacy for HPSC.^15^^,^^33^^,^^34^ The HPSC-NAT will help policy makers gain an overview of current government policy creation and implementation and leverage actions directly or indirectly to focus on local sports clubs. The process of monitoring government support to promote healthful policies has shown success in other domains, such as policies pertaining to food environments.^35^ With consistent use of this audit tool, it is possible to monitor country progress in developing national policies to support HPSC, compare regional differences in policy development and share knowledge on the health benefits obtained by implementing HPSC polices.

The multiple steps and rigorous methods used to create the HPSC-NAT were essential to produce an evidence-informed and contextually relevant tool to monitor HPSC policies at a national level. Having a project team of international experts, researchers and academics brought diversity to the development process^29^ while using participatory research methods to directly involve stakeholders to develop the final tool added universal understanding of each section and increases the likelihood for adoption and usability.^36^

Several limitations to this study should be noted. First, although the rapid literature review produced over 250 initial indicators, more indicators may have been found through a more comprehensive systematic review. Second, the audit tool was created in English. This is not the first language of many countries in which it will be used and a translation process is required for further development. Third, an extensive pilot data collection and analysis using the HPSC-NAT would support more comprehensive testing of the usefulness and acceptability of this tool. Testing of the audit tool is currently taking place in two countries, Ireland and France, and planned for additional countries.

Completing a policy review using the HPSC-NAT will provide comprehensive overviews of current policies, strategies and action plans supporting HP in organized sports clubs at a national level. This will enable country-level surveillance to evaluate gaps between national policies and local-level implementation, monitor changes and development of supportive HP policies, compare government policies and share knowledge between sectors and countries. Identifying national policies will help sports clubs to set criteria for promoting health within their own setting and community. Many sports clubs are voluntary by nature and therefore need government action, resources and a support system to make sustainable HP changes. The HPSC-NAT can help build a solid knowledge base to^1^ identify potential gaps and barriers,^2^ learn from experience^3^ monitor progress,^4^ understand challenges for future policy framing and planning in this context and^5^ build understanding of the HPSC approach.

## Funding

The original work on which the article is based was supported by the WHO Regional Office for Europe and funded by a partnership on the PROSCeSS project between Santé publique France, Université de Lorraine and Université Côte d’Azur.

*Disclaimer*: The authors take sole responsibility for the content of this article. S.W. is a staff member of the WHO Regional Office for Europe. The author affiliated with the World Health Organization (WHO) is alone responsible for the views expressed in this publication and they do not necessarily represent the decisions or policies of the WHO.

Key points

- Organized sports clubs are an ideal setting to promote the physical, mental and social health of stakeholders and the surrounding community.
- Organized sports clubs often rely on external policy support from national governments and affiliated sports federations to implement HP actions.
- There is a gap in our knowledge of the national policies that exist to support sports clubs to promote health beyond the provision of sport and competition.
- The health promoting sports club-national audit tool (HPSC-NAT) was created through a rigorous process using international experts and stakeholders in fields of sports and physical activity.
- To help determine policies and action plans at the national government level, the HPSC-NAT includes the following 11 sections: Role of ministry or department, Policies, Communication, Implementation and Dissemination, Evaluation and Measurement methods, Sub-national-level policies, Funding and Coordination, Participative approach, Actors and Stakeholders, National sporting events, Case studies and HPSC-NAT implicated stakeholders.
